# Simultaneous estimation of a model-derived input function for quantifying cerebral glucose metabolism with [^18^F]FDG PET

**DOI:** 10.1186/s40658-024-00614-6

**Published:** 2024-01-29

**Authors:** Lucas Narciso, Graham Deller, Praveen Dassanayake, Linshan Liu, Samara Pinto, Udunna Anazodo, Andrea Soddu, Keith St Lawrence

**Affiliations:** 1 Brain Health Imaging Centre, Centre for Addiction and Mental Health, Toronto, ON Canada; 2 Department of Psychiatry, University of Toronto, Toronto, ON Canada; 3 Imaging Program, Lawson Health Research Institute, 268 Grosvenor St, London, ON N6A 4V2 Canada; 4 Department of Medical Biophysics, Western University, London, ON Canada; 5 Department of Biomedical Gerontology, PUCRS, Porto Alegre, Rio Grande do Sul Brazil; 6 Neurology and Neurosurgery, Montreal Neurological Institute, McGill University, Montreal, QC Canada; 7 Department of Physics and Astronomy, Western University, London, ON Canada

**Keywords:** [^18^F]FDG, Cerebral metabolic rate of glucose, Image-derived input function, Microparameters, Model-derived input function, Non-invasive measurement, PET, Simultaneous estimation

## Abstract

**Background:**

Quantification of the cerebral metabolic rate of glucose (CMRGlu) by dynamic [^18^F]FDG PET requires invasive arterial sampling. Alternatives to using an arterial input function (AIF) include the simultaneous estimation (SIME) approach, which models the image-derived input function (IDIF) by a series of exponentials with coefficients obtained by fitting time activity curves (TACs) from multiple volumes-of-interest. A limitation of SIME is the assumption that the input function can be modelled accurately by a series of exponentials. Alternatively, we propose a SIME approach based on the two-tissue compartment model to extract a high signal-to-noise ratio (SNR) model-derived input function (MDIF) from the whole-brain TAC. The purpose of this study is to present the MDIF approach and its implementation in the analysis of animal and human data.

**Methods:**

Simulations were performed to assess the accuracy of the MDIF approach. Animal experiments were conducted to compare derived MDIFs to measured AIFs (*n* = 5). Using dynamic [^18^F]FDG PET data from neurologically healthy volunteers (*n* = 18), the MDIF method was compared to the original SIME-IDIF. Lastly, the feasibility of extracting parametric images was investigated by implementing a variational Bayesian parameter estimation approach.

**Results:**

Simulations demonstrated that the MDIF can be accurately extracted from a whole-brain TAC. Good agreement between MDIFs and measured AIFs was found in the animal experiments. Similarly, the MDIF-to-IDIF area-under-the-curve ratio from the human data was 1.02 ± 0.08, resulting in good agreement in grey matter CMRGlu: 24.5 ± 3.6 and 23.9 ± 3.2 mL/100 g/min for MDIF and IDIF, respectively. The MDIF method proved superior in characterizing the first pass of [^18^F]FDG. Groupwise parametric images obtained with the MDIF showed the expected spatial patterns.

**Conclusions:**

A model-driven SIME method was proposed to derive high SNR input functions. Its potential was demonstrated by the good agreement between MDIFs and AIFs in animal experiments. In addition, CMRGlu estimates obtained in the human study agreed to literature values. The MDIF approach requires fewer fitting parameters than the original SIME method and has the advantage that it can model the shape of any input function. In turn, the high SNR of the MDIFs has the potential to facilitate the extraction of voxelwise parameters when combined with robust parameter estimation methods such as the variational Bayesian approach.

**Supplementary Information:**

The online version contains supplementary material available at 10.1186/s40658-024-00614-6.

## Background

Positron emission tomography (PET) imaging with 2-deoxy-2-[^18^F]fluoro-D-glucose ([^18^F]FDG) has proven valuable for assessing cerebral energy metabolism in neurological diseases [1, 2]. Quantification of dynamic [^18^F]FDG PET data is typically performed by measuring the cerebral metabolic rate of glucose (CMRGlu) by means of Patlak graphical analysis [3, 4], which requires measuring the time-varying concentration of [^18^F]FDG in plasma (i.e., the arterial input function or AIF). Non-invasive alternatives to arterial sampling have primarily focused on extracting an image-derived input function (IDIF). PET-only methods of extracting the IDIF from the carotid arteries require careful correction of partial volume effects (PVE) [5, 6], which is typically performed by measuring the point-spread function of the PET system at the measurement location [7]. PVE corrections can be facilitated by combining magnetic resonance imaging (MRI) angiography of the feeding arteries [8], but PET and MRI misalignments can introduce errors in the IDIF—which require sophisticated registration methods [9]—and complex vessel segmentation approaches can limit applicability [10]. Alternatively, hybrid PET/MR imaging allows for simultaneous acquisition of both functional and anatomical information, reducing misalignment errors [11, 12] and facilitating PVE correction [13, 14]. Although these approaches are promising, their complexity hinders the widespread use of IDIFs when evaluating [^18^F]FDG PET images [15]. Lastly, acquiring dynamic [^18^F]FDG PET data with a large axial field-of-view scanner may facilitate the localization of large arteries needed to extract the IDIF [16]; however, the limit access to such scanners fosters the development of simpler and readily available techniques to be implemented in any PET centre.

An alternative to extracting the IDIF from feeding arteries is the simultaneous estimation (SIME) approach. By assuming the input function is the same for all brain regions, the SIME method models the IDIF as a series of exponentials by which parameters are obtained by simultaneously fitting time-activity curves (TACs) from various volumes-of-interest (VOIs) and using blood samples as scalers [17–19]. Although promising, modelling the input function as a series of exponentials may not always be accurate [20], especially for the initial period following injection [21]. Strategies to overcome this limitation include maintaining a uniform experimental design across subjects and imaging sites (e.g., fixed injection duration), better characterizing the peak by incorporating the injection duration into the model [22], modelling the pre-peak phase as a straight line [23], and using the IDIF first pass extracted with an MR-based method as a prior [24, 25].

This study investigated an alternative SIME approach in which the input function is defined by the irreversible two-tissue compartment model (2TCM) used to characterize dynamic [^18^F]FDG PET data, rather than by a series of exponentials. The model-derived input function (MDIF) is derived from the whole-brain (WB) TAC, given it has the highest signal-to-noise ratio (SNR) obtainable in the PET brain data, with rate constants obtained by SIME. This approach requires fewer fitting parameters to define the input function compared to the original SIME approach, although late time-point blood samples are still used as anchors as recommended when extracting IDIFs [6, 15]. The MDIF is free of the typical PVE that affect the IDIFs extracted from the feeding arteries and achieves low noise levels, while still retaining the subject- and study-specific shape of the input function, making the approach readily available and suitable for any experimental design and injection protocol. The overall objective of this work was to investigate the performance of the MDIF SIME approach.

## Materials and methods

### Model-derived input function

PET imaging of cerebral glucose metabolism with [^18^F]FDG is typically based on the irreversible two-tissue compartment model [3], in which [^18^F]FDG enters the tissue via glucose transporters defined by an influx rate constant ${K}_{1}$ (in mL/g/min). Once in the first compartment (i.e., the free pool), [^18^F]FDG can either return to the blood pool at an efflux rate defined by ${k}_{2}$ (in min^−1^) or be phosphorylated at a rate defined by ${k}_{3}$ (in min^−1^) and subsequently trapped in the metabolic pool. These processes are defined by the following two differential equations:

1 $$\frac{d}{dt}{C}_{1}\left(t\right)={K}_{1}{C}_{p}\left(t\right)-\left({k}_{2}+{k}_{3}\right){C}_{1}\left(t\right)$$

2 $$\frac{d}{dt}{C}_{2}\left(t\right)={k}_{3}{C}_{1}\left(t\right)$$

where ${C}_{1}\left(t\right)$ represents the activity concentration in the free pool and ${C}_{2}\left(t\right)$ in the metabolic pool. Eq. (3) provides the solution to Eqs. (1) and (2) for the total measured activity (i.e., ${C}_{PET}\left(t\right)=\left(1-{V}_{b}\right)\left({C}_{1}\left(t\right)+{C}_{2}\left(t\right)\right)+{V}_{b}{C}_{b}\left(t\right)$), which includes an additional term ${V}_{b}$ (in mL/g) to account for blood-borne [^18^F]FDG activity [26]:

3 $${C}_{PET}\left(t\right)=\left(1-{V}_{b}\right)\frac{{K}_{1}}{{k}_{f}}\left({k}_{3}+{k}_{2}{e}^{-{k}_{f}t}\right)*{C}_{p}\left(t\right)+{V}_{b}{C}_{b}\left(t\right)$$

where $*$ represents the convolution operation; ${C}_{p}\left(t\right)$ and ${C}_{b}\left(t\right)$ represent the plasma and whole-blood activity concentration of [^18^F]FDG, respectively; and ${k}_{f}={k}_{2}+{k}_{3}$.

The previous equations can be combined and rearranged to derive an expression for the input function, i.e., the MDIF (see Appendix for full derivation). By selecting the WB TAC (${C}_{wb}\left(t\right)$) to define the MDIF, considering it has the highest SNR obtainable in a dynamic PET brain image, and assuming ${C}_{b}\left(t\right)=R{C}_{p}\left(t\right)$ (where $R$ is the blood-to-plasma ratio), the MDIF is given by:

4 $${C}_{p}^{MDIF}\left(t\right)=\frac{1}{R{V}_{b}}\left[{C}_{wb}\left(t\right)+\frac{1-{V}_{b}}{R{V}_{b}}{K}_{1}\left(\frac{{k}_{3}-{\alpha }_{2}}{{\alpha }_{2}-{\alpha }_{1}}{e}^{-{\alpha }_{2}t}-\frac{{k}_{3}-{\alpha }_{1}}{{\alpha }_{2}-{\alpha }_{1}}{e}^{-{\alpha }_{1}t}\right)*{C}_{wb}\left(t\right)\right]$$

where ${\alpha }_{\mathrm{1,2}}=a\mp \sqrt{{a}^{2}-b}$, with $a=\frac{1}{2}\left(\frac{1-{V}_{b}}{R{V}_{b}}{K}_{1}+{k}_{f}\right)$ and $b=\frac{1-{V}_{b}}{R{V}_{b}}{K}_{1}{k}_{3}$.

By employing this approach, the input function is defined by the four model parameters (${K}_{1}, {k}_{2},{k}_{3}$ and ${V}_{b}$) that characterize the WB TAC. Eq. (4) was developed for [^18^F]FDG given it obeys the irreversible 2TCM and follows two key assumptions. First, [^18^F]FDG does not require metabolite correction as the tracer and its metabolites are trapped in the cell and clearance of [^18^F]FDG-6-phosphate is slow for acquisition times of up to 60 min [27]. Second, the blood-to-plasma ratio was assumed to be a constant value for simplification purposes [28], although this assumption might not be valid for non-primates [29]. The appendix provides the general solution for reversible radiotracers. We are currently developing a variation of the MDIF method for tracers that require metabolite correction, but this is beyond the scope of the current work.

### MDIF SIME implementation

The SIME procedure [17] requires TACs from multiple volumes-of-interest (VOIs) to derive the whole-brain kinetic parameters that define the MDIF (Eq. (4)). These TACs can be from anatomical VOIs or grouped based on functional similarity, as previously suggested by Wong et al., to identify TACs with distinct kinetics [18]. We implemented a *k*-means clustering algorithm (explained below) to group TACs by functional similarity. After initial investigation, we observed that 3 clusters of each tissue type (i.e., grey [GM] and white matter [WM]) were sufficient to derive the MDIF, with little-to-no improvement observed by using more than 6 TACs (results not shown). A diagram outlining the MDIF SIME implementation is shown in Additional file 1: Fig. S1.

The parameters needed to compute the MDIF using Eq. (4) were obtained by simultaneously fitting six TACs to the irreversible 2TCM solution (Eq. (3)) using a non-linear least squares (NLLS) fitting routine. The algorithm runs until the cost function, comprising of the residual sum of squares (RSS; in Eq. (5)), is minimized and the WB model parameters used to define the MDIF is obtained.

5 $$RSS=\sum_{j=1}^{n}\sum_{i=1}^{T}{\left({C}_{j}^{fit}\left({t}_{i}\right)-{C}_{j}\left({t}_{i}\right)\right)}^{2}+\sum_{k=1}^{2}{\left({C}_{k}^{MDIF}-{C}_{k}\right)}^{2}$$

where ${C}_{j}\left(t\right)$ is the [^18^F]FDG-PET activity concentration of the *j*^th^ VOI, ${C}_{j}^{fit}\left(t\right)$ is the corresponding model estimate, $n$ is the number of VOIs (not including the WB TAC), and $T$ is the number of time-frames. Given the need for scalers (or anchors) to accurately obtain an input function with SIME [15], our procedure included two late time-point blood samples to act as anchors. ${C}_{k}$ is the *k*^th^ blood sample activity concentration, and ${C}_{k}^{MDIF}$ is given by Eq. (4) at the same time as the blood sample.

The fitting procedure was performed in MATLAB (The MathWorks Inc., R2023a) using the optimization routine *lsqnonlin* and four parameters per TAC were included in the fitting routine (${K}_{1}$, ${k}_{2}$, ${k}_{3}$, and ${V}_{b}$, for a total of 28 parameters). The upper bounds were set to 0.2 mL/g/min, 0.4 min^−1^, 0.2 min^−1^, and 0.10 mL/g for ${K}_{1}$, ${k}_{2}$, ${k}_{3}$, and ${V}_{b}$, respectively (based on literature values, see Additional file 1: Table S1). All lower bounds were set to 0.01. A constraint was added to the nonlinear optimization function implemented in this study to ensure the distribution volume (= ${K}_{1}/\left({k}_{2}+{k}_{3}\right)$) is less than unity (i.e., ${K}_{1}<{k}_{2}+{k}_{3}$), which was based on literature values (Additional file 1: Table S1).

#### *k-means* clustering

For the animal and human studies, VOIs were generated by clustering the TACs based on functional similarity by implementing a *k*-means clustering algorithm (Euclidean distance, 500 iterations, 10 replicates) that used the *k*-medoids MATLAB function *kmediods* [30]. For clustering only, PET images were denoised by applying the 3D highly constrained backprojection (HYPR3D) method [31], in which a Gaussian filter kernel of standard deviation 3 voxels was used (equivalent to 6.3 × 6.3 × 6.1 mm^3^ for the human PET data used in this study). Although HYPR3D introduces bias to the dynamic signal, which is known to affect quantification [32], this bias is not expected to influence the *k*-means clustering algorithm. Instead, denoising with HYPR3D improves the clustering of similar TACs. Additional *k*-means clustering implementation details are given in the appropriate sections below.

### IDIF SIME implementation

For comparison to our MDIF SIME method, the original SIME approach based on the work by Feng et al. and Wong et al. [17, 18] was implemented to extract an IDIF by estimating its 7 parameters (Eq. (6)), while simultaneously fitting six VOI TACs (WB TAC not included) using a NLLS fitting routine. The same cost function from the MDIF SIME was used (Eq. (5)), except for an additional weight (= 10) included in the blood samples portion of Eq. (5). All fitting was performed in MATLAB using the optimization routine *lsqnonlin* and four parameters per TAC were included in the fitting routine (${K}_{1}$, ${k}_{2}$, ${k}_{3}$, and ${V}_{b}$; same constraint and bounds as described above) in addition to the 7 parameters required for the IDIF (Eq. (6)): ${A}_{i}$, ${\lambda }_{i}$, with *i* = 1, 2, 3, and a delay term $\delta$ (total of 31 parameters). Upper bounds were 4000 kBq/mL/min, 100 kBq/mL, 50 kBq/mL, 25 min^−1^, 1 min^−1^, and 0.1 min^−1^, for ${A}_{1}$, ${A}_{2}$, ${A}_{3}$, ${\lambda }_{1}$, ${\lambda }_{1}$, and ${\lambda }_{3}$, respectively. Lower bounds were set to zero. The delay term ($\delta$) was set to vary within ± 10 s of an initial delay computed a priori based on the rise of the WB TAC.

6 $${C}_{p}^{IDIF}\left(t\right)=({A}_{1}\left(t-\delta \right)-{A}_{2}-{A}_{3}){e}^{-{\lambda }_{1}\left(t-\delta \right)}+{A}_{2}{e}^{-{\lambda }_{2}\left(t-\delta \right)}+{A}_{3}{e}^{-{\lambda }_{3}\left(t-\delta \right)}$$

### Standalone fitting routine

To extract microparameters from anatomical VOIs, each TAC was fit to the irreversible 2TCM solution (Eq. (3)) using the MATLAB optimization routine *lsqnonlin*. Four parameters were included in the weighted NLLS (WNLLS) fitting routine (${K}_{1}$, ${k}_{2}$, ${k}_{3}$, and ${V}_{b}$). Upper bounds were set to 0.5 mL/g/min, 0.5 min^−1^, 0.2 min^−1^, and 1 mL/g for ${K}_{1}$, ${k}_{2}$, ${k}_{3}$, and ${V}_{b}$, respectively. All lower bounds were set to zero. The cost function was defined as a weighted RSS (WRSS), given by $WRSS=\sum_{i=1}^{T}{w}_{i}{\left({C}_{j}^{fit}\left({t}_{i}\right)-{C}_{j}\left({t}_{i}\right)\right)}^{2}$. Weights for the fitting (${w}_{i}$) were defined as the inverse of variance of the PET measurement error (i.e., the ratio between the time-frame duration and the WB activity concentration, normalized to the maximum weight) [33]. A brain density of 1.05 mL/g was used throughout the analyses.

### Simulations

**Fig. 1:**
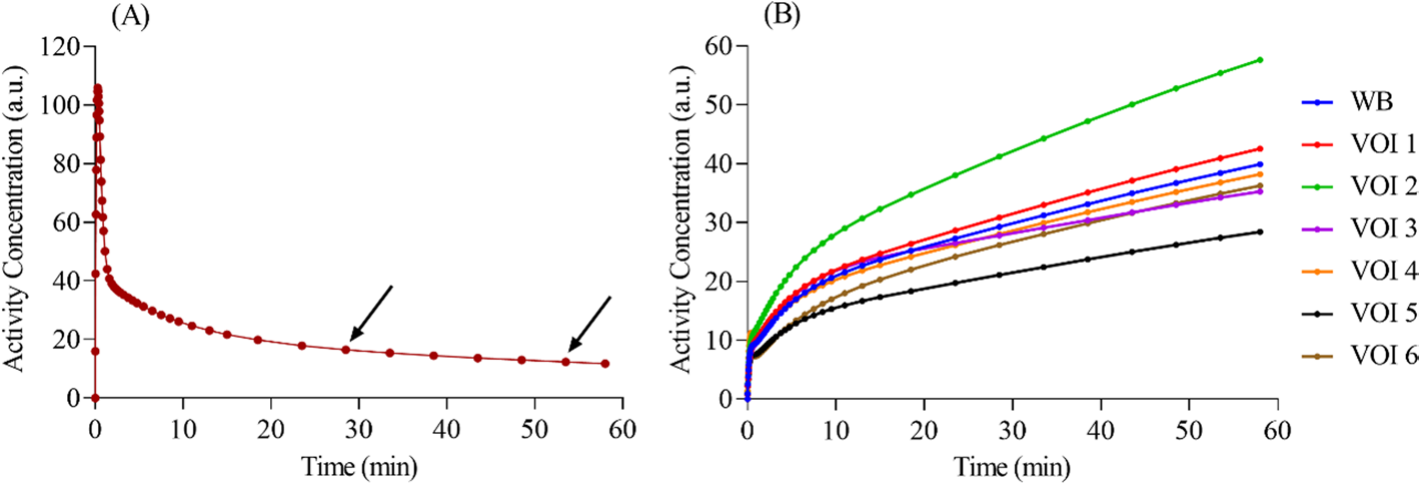
Theoretical **A** AIF and **B** TACs used in the simulations. Curves were interpolated to the time frames used in the human experiments (symbols). The arrows represent the scalers used in the SIME simulations. AIF: Arterial input function; TAC: Time-activity curve; VOI: Volume-of-interest; WB: Whole brain

A theoretical AIF (${C}_{p}\left(t\right)$) was generated by Eq. (6) using ${A}_{1}$ = 850 (in arbitrary units [a.u.]/min), ${A}_{2}$ = 22 and ${A}_{3}$ = 21 (in a.u.), ${\lambda }_{1}$ = 4, ${\lambda }_{2}$ = 0.12 and ${\lambda }_{3}$ = 0.01 (in min^−1^), and $\delta$ = 0. These constants were obtained from Feng et al. and are based on experimental AIF measurements from a human study [34]. The theoretical AIF was used in the irreversible 2TCM solution (Eq. (3)) to generate simulated regional TACs for six theoretical VOIs (Table 1). The microparameters shown in Table 1 were chosen from a variety of studies [9, 35–37] and reflect the need for TACs with distinct kinetics for the SIME approach [18, 38]. The net clearance rate constant of [^18^F]FDG (${K}_{i}$ = ${K}_{1}{k}_{3}/{k}_{f}$, in mL/g/min), is also shown in Table 1. A theoretical WB TAC was obtained by using the average microparameters from the six VOIs. Fig. 1 shows the theoretical AIF alongside simulated TACs. These curves and microparameters were considered the ground-truth for the simulations. Lastly, simulations used anchors extracted from the theoretical AIF at 28.5 and 53.5 min timepoints, as indicated by the arrows in Fig. 1A.

**Table 1:** Microparameters used to define regional TACs for six theoretical VOIs included in the simulations

Microparameters	VOI 1^a^	VOI 2^b^	VOI 3^c^	VOI 4^d^	VOI 5^a^	VOI 6^d^	Average (WB)^e^
${K}_{1}$(mL/g/min)	0.105	0.123	0.096	0.088	0.069	0.062	0.090
${k}_{2}$(min^−1^)	0.148	0.121	0.109	0.123	0.129	0.071	0.117
${k}_{3}$(min^−1^)	0.074	0.079	0.042	0.071	0.064	0.067	0.066
${V}_{b}$(mL/g)	0.050^f^	0.059	0.050^f^	0.084	0.050^d^	0.050	0.067
${K}_{i}$(mL/g/min)	0.035	0.049	0.027	0.032	0.023	0.030	0.033

^a^Based on the work of Reivich et al. [35]

^b^Based on the work of Sari et al. [9]

^c^Based on the work of Zanotti-Fregonara et al. [36]

^d^Based on the work of Huisman et al. [37]

^e^WB TAC was obtained by using the average microparameters

^f^CBV was not reported

*CBV* Cerebral blood volume; *TACs* Time-activity curve; *VOI* Volume-of-interest; *WB* Whole brain

To confirm the accuracy of the MDIF SIME routine, errors due to estimating the WB microparameters (Eq. (4)) with the SIME routine were evaluated. The MDIF was generated as described above and used to fit the six VOI TACs (Table 1) to the irreversible 2TCM solution (Eq. (3)) in the standalone fitting routine. Then, the best-fit estimates of the four parameters (${K}_{1}$, ${k}_{2}$, ${k}_{3}$, and ${V}_{b}$) were compared to their input values.

### Animal study

The MDIFs were assessed in comparison to AIFs using retrospective data from animal experiments [12] collected at the Lawson Health Research Institute. All experiments were conducted according to the regulations of the Canadian Council on Animal Care and approved by the Animal Care Committee at Western University. Data from juvenile Duroc pigs were collected on a 3 T hybrid PET/MR scanner (Biograph mMR, Siemens Heathineers, Erlangen, Bavaria, Germany) using a 12-channel PET-compatible receiver head coil. The five animals included in this study (weight, 21 ± 2 kg; injected [^18^F]FDG activity, 90 ± 20 MBq; blood glucose level, 5.0 ± 1.6 mmol/L) were scanned under an anaesthetic combination of 1–3% isoflurane and 6–25 mL/kg/h intravenous infusion of propofol. [^18^F]FDG was administered via a cephalic vein immediately followed by a 60-min acquisition of PET data in list-mode. Arterial blood was continuously withdrawn from a femoral artery using an automated MR-compatible system (Swisstrace GmbH, Menzingen, Canton of Zug, Switzerland) at a sampling rate of 4 mL/min for the first 5 min, 1 mL/min for the next 5 min, and 0.5 mL/min for the remaining 50 min. Arterial blood samples were processed and converted into AIFs via rebinning to match the reconstructed PET time frames using pSample (PMOD Technologies LLC). Each measured AIF was corrected for dispersion and delay [39].

Dynamic PET images were reconstructed offline with the Siemens e7 tools into 51 time-frames (15 × 2 s, 6 × 5 s, 8 × 15 s, 4 × 30 s, 5 × 60 s, 5 × 120 s, 8 × 300 s) using a 3-dimensional ordinary Poisson ordered subset expectation maximization (3D-OP-OSEM) method (3 iterations, 21 subsets) with corrections for decay, random coincidences, dead-time, detector normalization, data rebinning, attenuation, and scatter. Images were reconstructed into a 344 × 344 × 127 matrix with voxel size of 0.8 × 0.8 × 2.0 mm and smoothed with a 3D gaussian filter of 4 mm. Simultaneously to the PET acquisition, T_1_-weighted MR images were acquired (magnetization-prepared rapid gradient-echo sequence [MPRAGE]; repetition/echo times [TR/TE], 2000/2.98 ms; inversion time, 900 ms; field-of-view [FoV], 256 × 256 mm^2^; isotropic voxel size, 1 mm^3^; flip angle [α], 9°; 176 slices). At the end of the experiment, the animals were euthanized according to the animal care guidelines and transported to a computed tomography (CT) scanner to obtain a post-mortem CT-based attenuation correction map.

For the *k*-means clustering algorithm, a semi-automatic procedure was used to define a VOI encompassing the brain in each anatomical slice. The final WB VOI, which was the composite of all the slices, was used to generate seven clusters with the *k*-means clustering approach as described above. The cluster containing mostly non-brain regions (i.e., blood vessels and cerebrospinal fluid voxels) was excluded from the SIME algorithm. The SIME approach incorporated arterial samples collected at 20–30 min and 40–60 min post-injection as anchors. Although a constant blood-to-plasma ratio may not be valid for the porcine model used in this study [29], we assumed $R$ = 1 throughout the analysis of the animal data.

### Human study

Retrospective data from 18 neurologically healthy volunteers (44 ± 15 years, 77 ± 17 kg, 9 M/9F; average injected activity, 180 ± 40 MBq [range 130–260 MBq]; average injected activity per body weight, 2.43 ± 0.39 MBq/kg [range 1.80–3.70 MBq/kg]; blood glucose level, 5.0 ± 0.7 mmol/L [range 4.1–6.8 mmol/L]) collected at the Lawson Health Research Institute were used to assess the feasibility of obtaining MDIFs from dynamic [^18^F]FDG PET data. The study was approved by the Western University Health Sciences Research Ethics Board and was conducted in accordance with the Declaration of Helsinki ethical standards. Participants provided written informed consent in compliance with the Tri-Council Policy Statement of Ethical Conduct for Research Involving Humans.

Scanning was performed on a 3 T hybrid PET/MR scanner (Biograph mMR) using a 16-channel PET-compatible coil (12- and 4-channel head and neck coils, respectively). Each participant had their head immobilized during the 60-min list-mode PET acquisition performed following the [^18^F]FDG bolus injection, which was immediately followed by saline flush. PET data were reconstructed offline with the Siemens e7 tools into 50 time-frames (15 × 2 s, 6 × 5 s, 8 × 15 s, 4 × 30 s, 5 × 60 s, 3 × 120 s, 8 × 300 s, and 1 × 240 s) using the iterative reconstruction algorithm 3D-OP-OSEM (3 iterations, 21 subsets, 3D gaussian filter of 2 mm^3^, and a zoom factor of 2). Dynamic PET images were reconstructed into a 172 × 172 × 127 matrix with voxel size of 2.1 × 2.1 × 2.0 mm (FoV, 359 × 359 × 258 mm^3^). Corrections for decay, random coincidences, dead-time, detector normalization, data rebinning, attenuation, and scatter were performed. MR-based attenuation correction was performed with a vendor-provided ultrashort echo time MRI sequence (TR, 11.94 ms; TE, 0.07 and 2.46 ms; α, 10º; FoV, 300 × 300 × 300 mm^3^; voxel size, 1.6 × 1.6 × 1.6 mm^3^). Motion correction was applied to the time frames 24–50 (corresponding to 1.5 to 60 min) by realigning each frame to the mean image using statistical parametric mapping (v12, SPM12; https://www.fil.ion.ucl.ac.uk/spm/software/spm12/). Each session also included acquiring an MPRAGE image (TR/TE, 2400/2.25 ms; α, 8°; FoV, 205 × 205 mm^2^; 240 slices; voxel size, 0.8 × 0.8 × 0.8 mm^3^), which was coregistered to the PET space using SPM12.

The MPRAGE image was segmented into six tissue classes using SPM12. A WB mask was created by combining GM and WM tissue probability maps (80% threshold; cerebrospinal fluid voxels excluded). Five clusters for each tissue class (i.e., GM and WM) were generated by implementing the *k*-means clustering algorithm as described above. Out of the 5 clusters, the 3 largest ones were chosen for the SIME algorithms. Two venous samples collected during dynamic PET imaging (one 20–30 min post-injection and the other 45–60 min post-injection) were used as scalers when arterial-to-venous [^18^F]FDG equilibrium was assumed [6, 40]. The blood-to-plasma ratio was assumed to be ~ 0.9 ($R$ = 0.9) to derive the MDIF from the human data [28].

#### Variance of estimated ${{\varvec{K}}}_{{\varvec{i}}}$

Coefficient of variation (CV) of ${K}_{i}$ estimates obtained by fitting each cluster TAC to the 2TCM solution was computed as $CV\left(\%\right)=100\sqrt{{\sigma }^{2}}/{K}_{i}$, where the variance (${\sigma }^{2}$) was obtained from the error propagation rule: $${\sigma }^{2}=\left[\begin{array}{ccc}\frac{\partial {K}_{i}}{\partial {K}_{1}}& \frac{\partial {K}_{i}}{\partial {k}_{2}}& \frac{\partial {K}_{i}}{\partial {k}_{3}}\end{array}\right]\gamma \left({K}_{1},{k}_{2},{k}_{3}\right){\left[\begin{array}{ccc}\frac{\partial {K}_{i}}{\partial {K}_{1}}& \frac{\partial {K}_{i}}{\partial {k}_{2}}& \frac{\partial {K}_{i}}{\partial {k}_{3}}\end{array}\right]}^{T}$$, where the Hessian (covariance) matrix ($\gamma$) was approximated as $\gamma ={\left({J}^{T}J\right)}^{-1}WRSS/T$ ($J$ is the Jacobian matrix from the MATLAB function *lsqnonlin* and $T$ the number of time frames).

#### Goodness of fit

Following the derivation of the MDIF and IDIF with their respective SIME algorithms, goodness of fit of each input function was assessed via the WRSS from the standalone fitting routine. For this, each input function was used to fit the GM and WM TACs, and WRSS was calculated for the first 3 min of data, as well as for the entire 60 min of data. In addition, fitting to the irreversible 2TCM solution was evaluated qualitatively for the GM and WM TACs of one representative subject, as well as for TACs from two randomly selected GM and WM voxels.

#### Regional measurements

Anatomical MPRAGE images were normalized to the Montreal Neurological Institute (MNI; McGill University, Montreal, Quebec, Canada) space using SPM12 and the same deformation field was applied to the dynamic PET data. Average TACs were extracted for GM and WM (99% threshold; subject space), as well as twelve VOIs (frontal, occipital, parietal, and temporal lobes, as well as insula, cingulate, hippocampus, precuneus, caudate nucleus, putamen, thalamus, and cerebellum; MNI space) using the automated anatomical labelling atlas (Wake Forest University PickAtlas, http://fmri.wfubmc.edu/cms/software). The mask generated for the twelve VOIs was multiplied by the GM mask (80% threshold; MNI space) to extract the central portion of the VOIs, avoiding PVE at the boundaries.

Patlak analysis ($t>{t}^{*}$ = 20 min) was performed for each VOI, from which the slope (${K}_{i}$; in mL/100 g/min) was converted to CMRGlu (in µmol/100 g/min) by assuming a lumped constant of 0.52 [35] and incorporating measurements of the blood concentration of glucose (in µmol/mL).

Voxelwise parametric images of microparameters were generated by incorporating the MDIF into the variational Bayesian approach proposed by Castellaro et al. [33]. Specifically for this step, the *k*-means clustering algorithm described above was used to generate a functional atlas containing 40 clusters for each subject: 20 GM, 10 WM and 10 non-brain clusters, the latter including cerebrospinal fluid and voxels surrounding the brain. In the variational Bayesian fitting routine, each cluster TAC was fit to the irreversible 2TCM solution, with its rate constants used as priors for all intra-cluster voxels assuming a Gaussian distribution of possible values. Parametric maps of CMRGlu were computed from ${K}_{i}$ images (= ${K}_{1}{k}_{3}/\left({k}_{2}+{k}_{3}\right)$) [35]. Resulting parametric images (i.e., CMRGlu, ${K}_{1}$, ${k}_{2}$, ${k}_{3}$, and ${V}_{b}$) were normalized to the MNI space using SPM12.

### Statistics

Area under the curve (AUC) was used to assess the similarity between input functions. Percent error was calculated as $100\left(m-\widehat{m}\right)/\widehat{m}$, where $m$ and $\widehat{m}$ are the observed and expected values, respectively. Percent difference between two observed measurements ${m}_{1}$ and ${m}_{2}$ was computed as $100\left|{m}_{1}-{m}_{2}\right|/\overline{m }$, where $\overline{m }$ is the average measurement. A repeated measures two-way ANOVA was used to evaluate if estimates were affected by input function (MDIF vs. IDIF) and VOI; multiple comparisons tests were used to evaluate differences between MDIF and IDIF estimates. Linear regression was used to compare MDIF and IDIF results, from which the line-of-best fit was obtained alongside the 95% confidence intervals (CIs) for both the slope and intercept. Correlation was assessed by means of the Pearson correlation coefficient ($\rho$). All datasets were found to be normally distributed with normal Q-Q plots. Statistical tests were performed using GraphPad Prism (version 9, GraphPad Software, San Diego, California USA, www.graphpad.com). Statistical significance was defined by $\alpha$ = 0.05. Measurements are expressed in terms of mean ± one standard deviation alongside the CIs in square brackets when relevant.

## Results

### Simulations

**Fig. 2:**
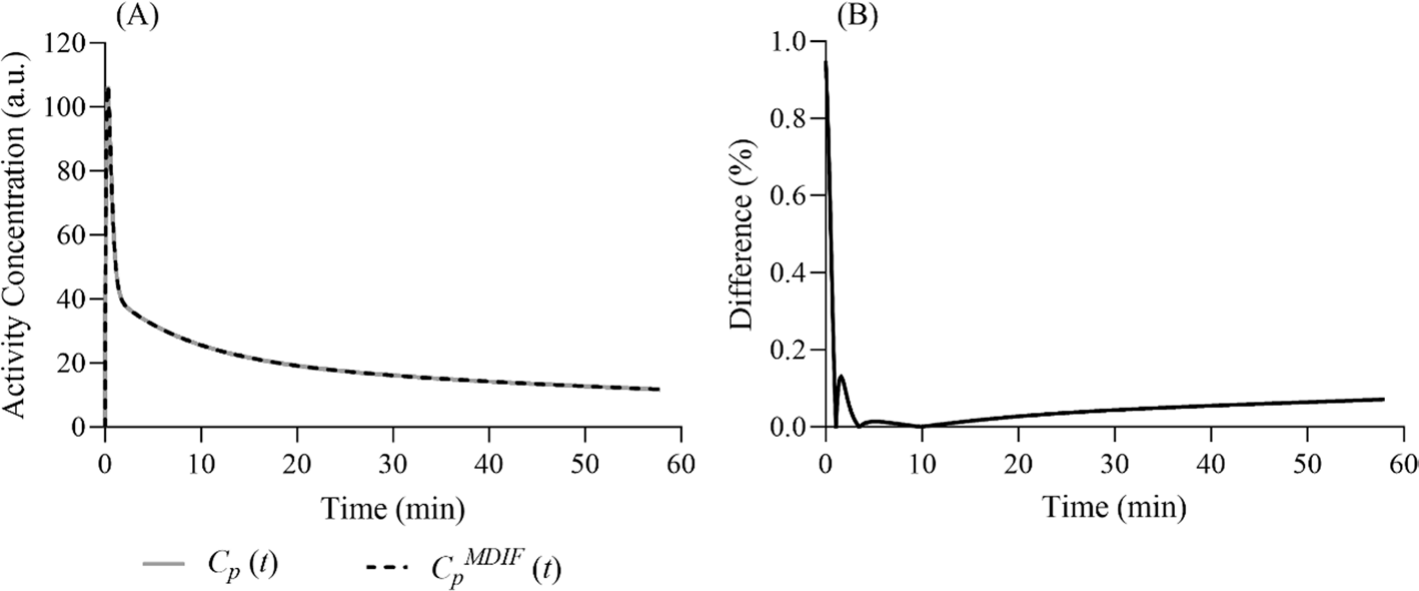
**A** True simulated input AIF (${C}_{p}\left(t\right)$; solid grey line) and MDIF (${C}_{p}^{MDIF}\left(t\right)$; dashed black line) obtained with the SIME algorithm. **B** Percent difference between MDIF and the true simulated AIF as a function of time. AIF: Arterial input function; MDIF: Model-derived input function; SIME: Simultaneous estimation; VOI: Volume-of-interest; WB: Whole brain

Figure 2A shows the MDIF obtained with the SIME algorithm and Fig. 2B the difference between the simulated (true) input function and the MDIF. Average error of microparameters were 0.33 ± 0.01% for ${K}_{1}$, 0.31 ± 0.07% for ${k}_{2}$, − 0.13 ± 0.04% for ${k}_{3}$, 0.32 ± 0.01% for ${V}_{b}$, and 0.05 ± 0.01% for ${K}_{i}$. These values were computed from the 6 VOIs used in the MDIF SIME approach. Regional ${K}_{i}$ estimates obtained with the MDIF (3.26 ± 0.89 mL/100 g/min) were nearly identical to their input values.

### Animal study

**Fig. 3:**
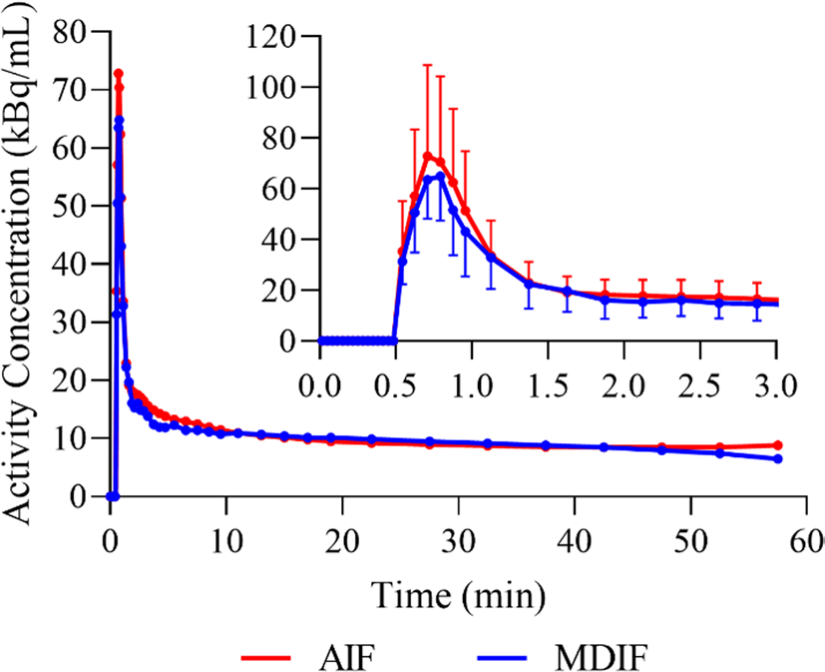
Comparison between average AIF (red) and MDIF (blue) for the five animals included in the study. Input functions were aligned to 0.5 min to compute each average curve. The inset shows the first 3 min of the two input functions including error bars that represent one standard deviation. AIF: arterial input function; MDIF: model-derived input function

Average WB estimates of ${K}_{1}$, ${k}_{2}$, ${k}_{3}$, and ${V}_{b}$ obtained using the AIF were 0.098 ± 0.021 mL/g/min, 0.148 ± 0.034 min^−1^, 0.013 ± 0.009 min^−1^, and 0.060 ± 0.019 mL/g, respectively (*n* = 5). Corresponding average WB ${K}_{i}$ was 0.86 ± 0.65 mL/100 g/min. Good MDIF-to-AIF agreement was observed across the five animals with an AUC ratio of 0.99 ± 0.06 (difference of 5.1 ± 2.2%; AUC_0-5_ = 0.93 ± 0.14, AUC_5-10_ = 0.92 ± 0.09, AUC_10-30_ = 1.05 ± 0.08, AUC_30-60_ = 0.99 ± 0.07, where the subscript refers to the time period in minutes). Percent difference between the arterial blood measurements used as anchors and the MDIF was 6.8 ± 4.1% for the 20–30-min anchor and 8.9 ± 6.6% for the 40–60-min anchor. Average WB estimates of the fitting parameters obtained using the MDIF were ${K}_{1}$ = 0.123 ± 0.028 mL/g/min (*p* = 0.048), ${k}_{2}$ = 0.195 ± 0.060 min^−1^ (*p* = 0.058), ${k}_{3}$ = 0.013 ± 0.004 min^−1^ (*p* = 0.958), and ${V}_{b}$ = 0.056 ± 0.016 mL/g (*p* = 0.691). No significant difference was observed in average WB ${K}_{i}$ estimates obtained with the MDIF (0.85 ± 0.48 mL/100 g/min, *p* = 0.911) compared to AIF measurements. Figure 3 shows a comparison between the average measured AIF and MDIF curves.

### Human study

**Fig. 4:**
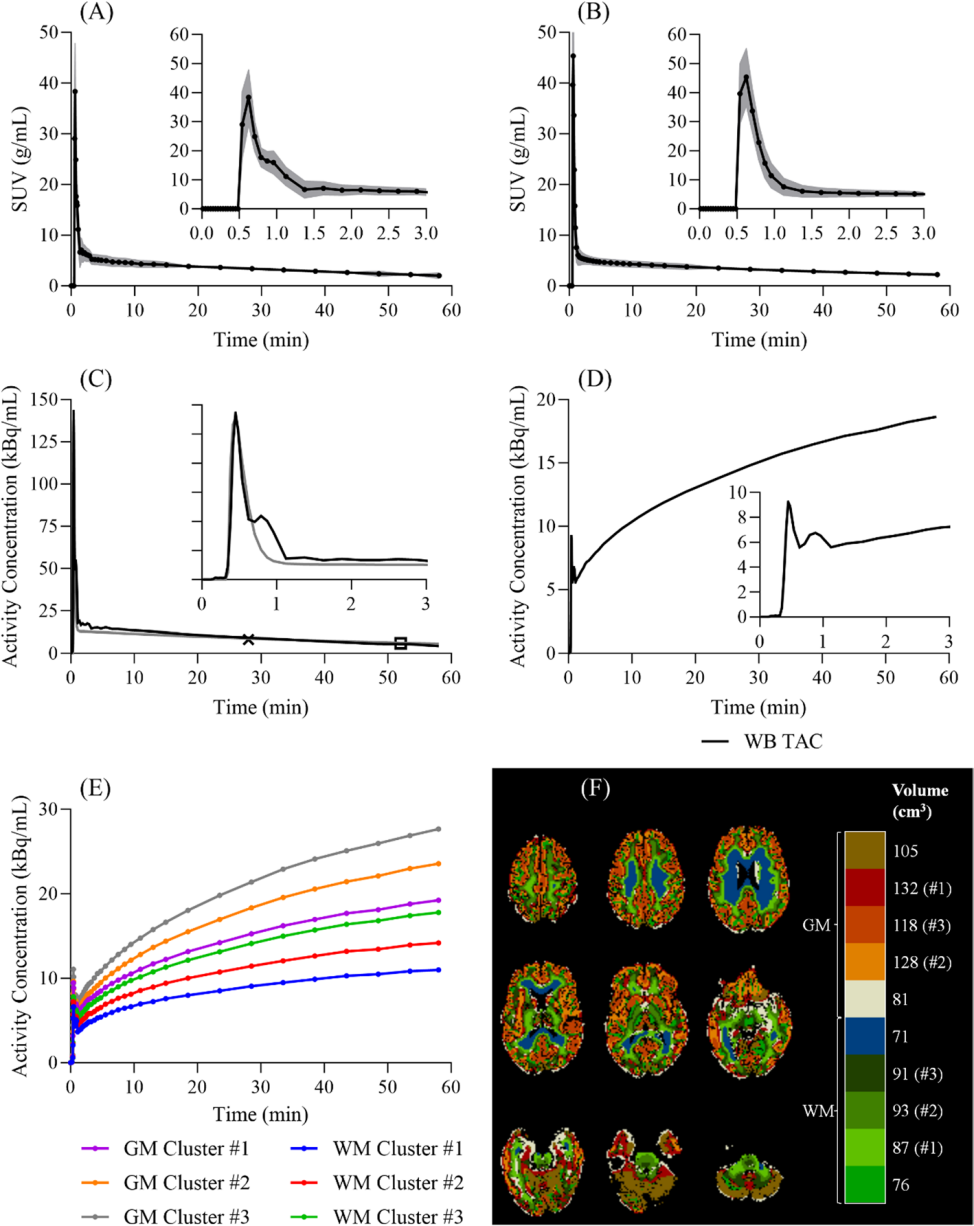
Average **A** MDIF and **B** IDIF curves obtained with their respective SIME approaches (*n* = 18). Grey-filled area on each graph represents ± one standard deviation. **C** SIME MDIF (black line) and SIME IDIF (solid grey line) from one representative subject. **D** WB TAC (solid black line) and **E** TACs from the six clusters used to obtain the MDIF and IDIF shown in **C**. The insets present the first 3 min of the respective curves. **F** Anatomical localization of the clusters whose TACs are shown in **E**. IDIF: image-derived input function. MDIF: Model-derived input function; TAC: Time-activity curve; GM: Grey matter; SIME: Simultaneous estimation; WB: Whole brain; WM: White matter

MDIFs were successfully extracted from all 18 subjects (average plasma curve is shown in Fig. 4A). Good overall agreement was observed between plasma MDIFs and IDIFs, with an AUC ratio of 1.02 ± 0.08 (difference of 6.3 ± 4.6%; AUC_0-5_ = 1.06 ± 0.24, AUC_5-10_ = 1.06 ± 0.20, AUC_10-30_ = 1.04 ± 0.10, AUC_30-60_ = 0.97 ± 0.05). Percent difference between the venous samples and the MDIFs was 4.2 ± 3.3% for the 20–30-min anchor (29.3 ± 2.6 min) and 9.3 ± 9.0% for the 45–60-min anchor (55.2 ± 1.7 min). Likewise, differences of 1.6 ± 2.0% and 2.4 ± 2.5% were found between the venous samples and the SIME IDIFs values at the two time points. Figure 4B presents the average plasma IDIF obtained with the original SIME approach (i.e., Eq. (6)). Figure 4C shows the plasma MDIF and IDIF (series of exponentials) from one representative subject, alongside the two venous samples used as anchors for all three input functions. In addition, Fig. 4D shows the corresponding WB [^18^F]FDG-TAC used to define the MDIF, Fig. 4E the cluster TACs used in both SIME procedures, and Fig. 4F the anatomical localization of the clusters presented in Fig. 4E. Average IDIF parameters obtained from the SIME routine were 2941 ± 1045 kBq/min, 6.6 ± 6.8 kBq, 9.2 ± 2.5 kBq, 10.1 ± 1.9 min^−1^, 0.32 ± 0.34 min^−1^, 0.012 ± 0.004 min^−1^, and 26 ± 7 s, for ${A}_{1}$, ${A}_{2}$, ${A}_{3}$, ${\lambda }_{1}$, ${\lambda }_{2}$, ${\lambda }_{3}$, and $\delta$, respectively.

The microparameters obtained from the two SIME approaches are summarized in Additional file 1: Table S2, alongside the CV values for each cluster. Average WB MDIF parameters were ${K}_{1}$ = 0.103 ± 0.019 mL/g/min, ${k}_{2}$ = 0.153 ± 0.055 min^−1^, ${k}_{3}$ = 0.050 ± 0.029 min^−1^, and ${V}_{b}$ = 0.067 ± 0.012 mL/g, with a CV of 0.4 ± 0.1%. For comparison, respective IDIF estimates from the standalone fitting routine were ${K}_{1}$ = 0.109 ± 0.024 mL/g/min (*p* = 0.473), ${k}_{2}$ = 0.145 ± 0.048 min^−1^ (*p* = 0.633), ${k}_{3}$ = 0.040 ± 0.012 min^−1^ (*p* = 0.095), and ${V}_{b}$ = 0.043 ± 0.010 mL/g (*p* < 0.001), with a CV of 4.9 ± 2.0%. Compared to MDIF SIME resulting clusters parameters, IDIF SIME results had higher estimates of ${K}_{1}$ (difference of approx. 9%) and ${k}_{2}$ (~ 4%), and lower estimates of ${k}_{3}$ (~ 16%) and ${V}_{b}$ (~ 30%), although significance was only observed for CBV (*p* < 0.001 for all clusters).

#### Goodness of fit

**Fig. 5:**
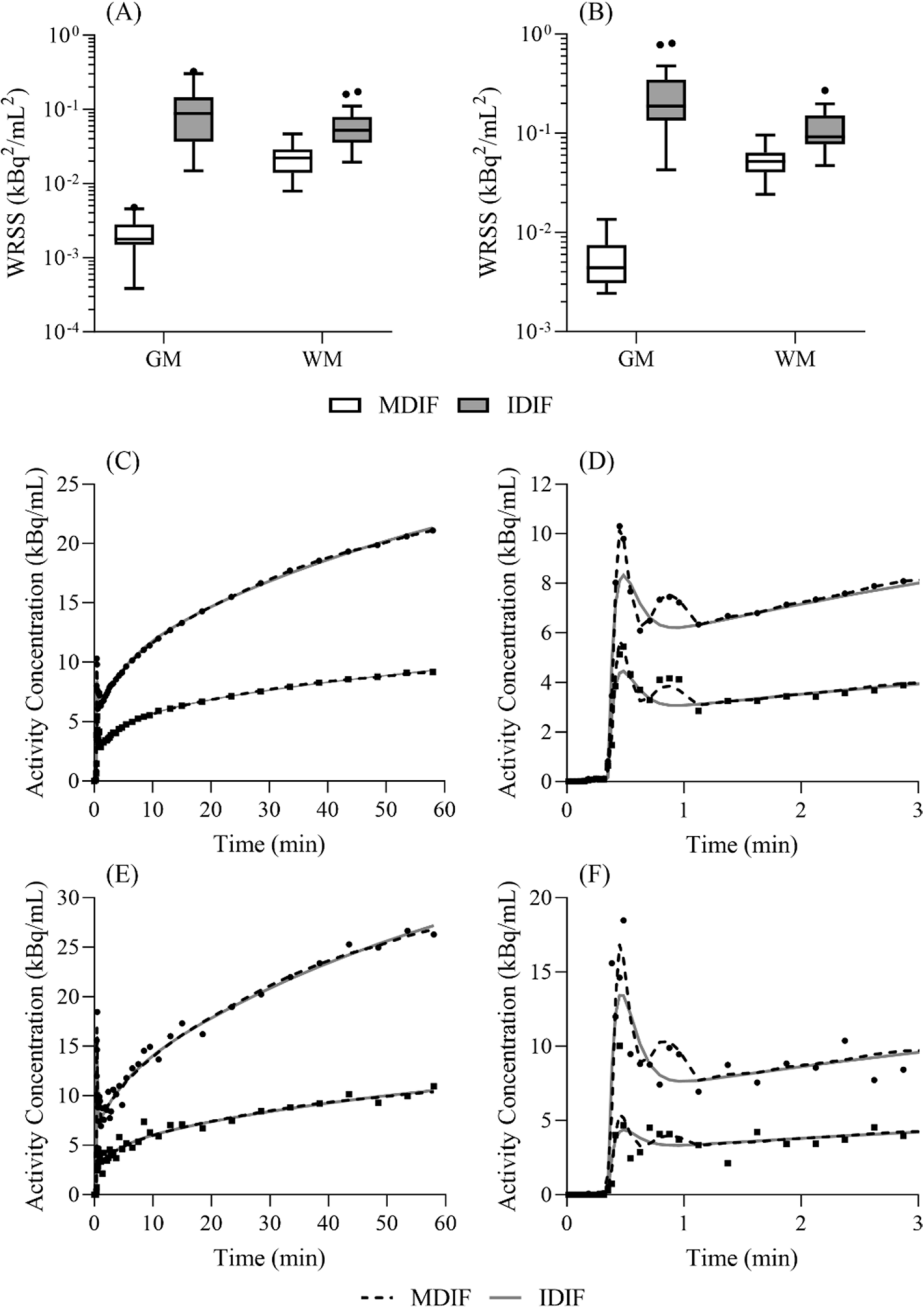
**A** and **B** Boxplots of WRSS obtained from the weighted square difference between measured and fitted curves obtained with the MDIFs (white-) and IDIFs (grey-filled rectangles). Results are shown for GM and WM TACs (*n* = 18). WRSS values are shown for **A** the first 3 min and **B** the complete 60 min of data. Logarithmic scale (base 10) was used for the *y*-axis. **C** Total GM (circles) and WM (squares) TACs together with the respective best-fit curves obtained with the MDIF (dashed black) and IDIF (grey) for the representative subject shown in Fig. 4. Fitted curves obtained with the MDIF presented a WRSS of 4.6 × 10^−3^ and 6.9 × 10^−2^ kBq^2^/mL^2^ for the GM and WM TACs, respectively. Respective fitted curves using the IDIF presented WRSS of 4.8 × 10^−1^ and 2.0 × 10^−1^ kBq^2^/mL^2^. **D** First 3 min of the TACs to visualize the initial pass of [^18^F]FDG. Similar graphs are shown in **E** and **F** for two randomly chosen voxels (one for each tissue type). Fitted curves obtained with the MDIF presented a WRSS of 7.2 and 2.8 kBq^2^/mL^2^ for the GM and WM voxel TACs, respectively. Respective fitted curves using the IDIF presented WRSS of 7.0 and 2.9 kBq^2^/mL^2^. [^18^F]FDG: 2-deoxy-2-[^18^F]fluoro-D-glucose; GM: grey matter; IDIF: Image-derived input function; MDIF: Model-derived input function; TAC: Time-activity curve; VOI: Volume-of-interest; WM: White matter; WRSS: weighted residual sum of squares

WRSS, indicating goodness of fit, for the first 3 min of each GM TAC was two orders of magnitude lower (*p* < 0.001) for the TACs fit using the MDIFs compared to corresponding TACs fit using the IDIFs (Fig. 5A). Likewise, a significantly lower WRSS for curves fitted with the MDIFs was also observed for the entirety of data (*p* < 0.001; Fig. 5B). Average WRSS for the first 3 min of data was (2.1 ± 1.2) × 10^−3^ and (1.1 ± 0.9) × 10^−1^ (kBq/mL)^2^ for the GM TACs fitted with the MDIFs and IDIFs, respectively (*n* = 18); corresponding average WRSS for the 60 min of data was (5.6 ± 3.3) × 10^−3^ and (2.7 ± 2.3) × 10^−1^ (kBq/mL)^2^. The stronger goodness of fit obtained with the MDIF is also demonstrated in Fig. 5C-F, which shows the fit of GM and WM TACs to the irreversible 2TCM solution. Results are for data from the same representative subject shown in Fig. 4.

#### Regional measurements

**Fig. 6:**
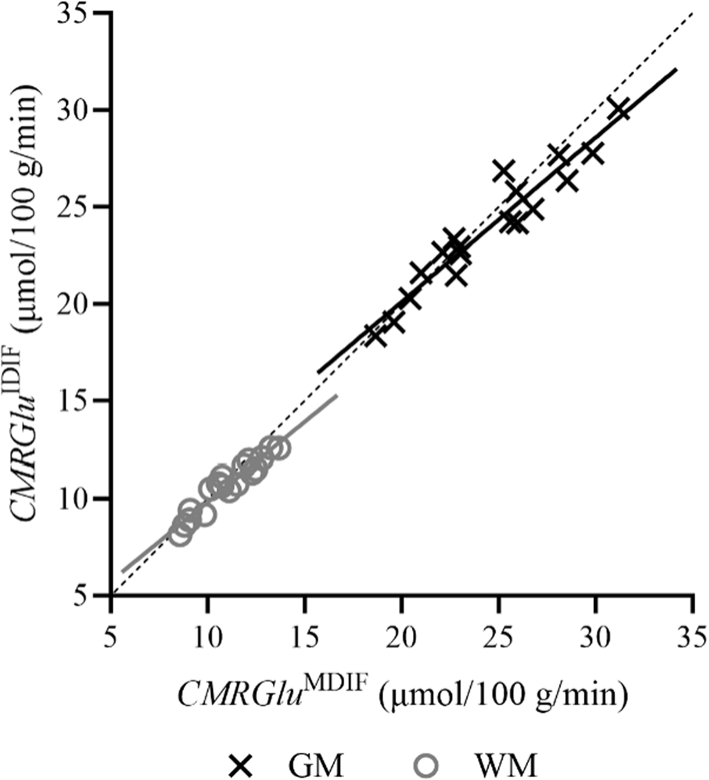
Linear regression comparing CMRGlu estimates from IDIF (*y*-axis) and MDIF (*x*-axis) for total GM (exes) and WM (circles) TACs extracted from healthy individuals (*n* = 18). Line-of-best fit is shown as a solid line. The identity line is represented by the dashed line. Average (± standard error) regression for GM had a slope of 0.85 ± 0.06 [0.71 to 0.98] and an intercept of 3.1 ± 1.6 µmol/100 g/min [− 0.2 to 6.5 µmol/100 g/min] (*R*^*2*^ = 0.92, $\rho$ = 0.96, *p* < 0.001). Similarly, average (± standard error) regression for WM had a slope of 0.82 ± 0.06 [0.69 to 0.95] and an intercept of 1.7 ± 0.7 µmol/100 g/min [0.2 to 3.1 µmol/100 g/min] (*R*^*2*^ = 0.92, $\rho$ = 0.96, *p* < 0.001). CMRGlu: Cerebral metabolic rate of glucose; GM: Grey matter; IDIF: Image-derived input function; MDIF: Model-derived input function; TAC: Time-activity curve; WM: White matter

**Fig. 7:**
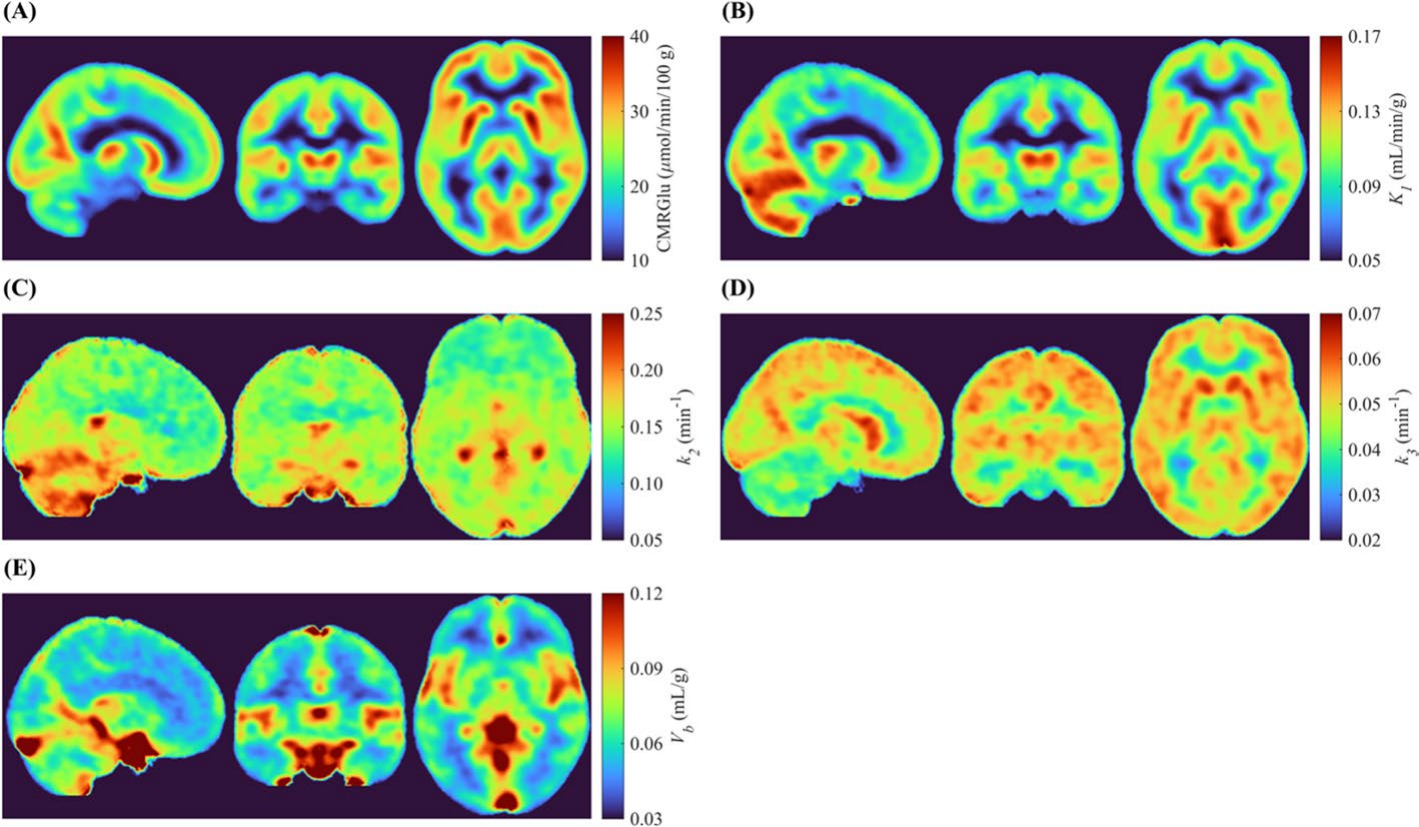
Groupwise (*n* = 18) **A** CMRGlu (in µmol/100 g/min) images, alongside **B** ${K}_{1}$ (in mL/g/min), **C** ${k}_{2}$ (in min^−1^), **D** ${k}_{3}$ (in min^−1^), and **E** ${V}_{b}$ (in mL/100 g) images, all generated by the variational Bayesian fitting routine. All images were generated using the MDIFs and normalized to the MNI space with SPM12

No significant differences between the two methods (i.e., MDIF and IDIF) were observed for either ${K}_{i}$ or CMRGlu estimates for anatomical VOIs, except for the cerebellum (*p* = 0.030 and *p* = 0.034 for ${K}_{i}$ and CMRGlu, respectively). Macro- and microparameter estimates for anatomical VOIs obtained with the MDIF are summarized in Table 2. For comparison, total GM and WM ${K}_{i}$ estimates obtained using the IDIFs were 2.51 ± 0.50 (*p* = 0.008) and 1.12 ± 0.22 mL/100 g/min (*p* = 0.139), respectively. Respective CMRGlu estimates were 23.9 ± 3.2 (*p* = 0.010) and 10.7 ± 1.3 µmol/100 g/min (*p* = 0.142). Figure 6 shows the linear regression comparing IDIF- and MDIF-derived CMRGlu estimates. Average (± standard error) regression for GM had a slope of 0.85 ± 0.06 [0.71–0.98] and an intercept of 3.1 ± 1.6 µmol/100 g/min [− 0.2 to 6.5 µmol/100 g/min] (*R*^*2*^ = 0.92, $\rho$ = 0.96, *p* < 0.001). Similarly, average (± standard error) regression for WM had a slope of 0.82 ± 0.06 [0.69 to 0.95] and an intercept of 1.7 ± 0.7 µmol/100 g/min [0.2 to 3.1 µmol/100 g/min] (*R*^*2*^ = 0.92, $\rho$ = 0.96, *p* < 0.001). Lastly, Fig. 7 shows average CMRGlu and microparameter images obtained with the variational Bayesian approach using MDIFs (*n* = 18); respective images obtained using the IDIFs are shown in Additional file 1: Fig. S2; differences between microparameters are shown in Additional file 1: Fig. S3.

**Table 2:** ${K}_{i}$ (mL/100 g/min) and CMRGlu (µmol/100 g/min) estimates obtained from Patlak graphical analysis alongside microparameters (${K}_{1}$ [mL/g/min], ${k}_{2}$ [min^−1^], ${k}_{3}$ [min^−1^], and ${V}_{b}$ [mL/g]) from the standalone fitting routine for the healthy individuals included in this study (*n* = 18)

VOI	${K}_{i}$	CMRGlu	${K}_{1}$	${k}_{2}$	${k}_{3}$	${V}_{b}$
Total GM	2.58 ± 0.56	24.5 ± 3.6	0.120 ± 0.022	0.160 ± 0.057	0.051 ± 0.029	0.072 ± 0.013
Total WM	1.16 ± 0.25	11.0 ± 1.6	0.054 ± 0.011	0.132 ± 0.041	0.040 ± 0.022	0.042 ± 0.007
Frontal lobe	3.06 ± 0.67	29.0 ± 4.3	0.115 ± 0.021	0.133 ± 0.054	0.055 ± 0.035	0.065 ± 0.012
Occipital lobe	2.65 ± 0.55	25.2 ± 3.5	0.114 ± 0.022	0.146 ± 0.052	0.053 ± 0.031	0.084 ± 0.013
Parietal lobe	2.76 ± 0.66	26.2 ± 4.5	0.111 ± 0.023	0.184 ± 0.055	0.042 ± 0.023	0.089 ± 0.019
Temporal lobe	2.64 ± 0.56	25.0 ± 3.6	0.115 ± 0.020	0.162 ± 0.069	0.055 ± 0.032	0.063 ± 0.013
Insula	2.70 ± 0.62	25.6 ± 4.1	0.107 ± 0.019	0.137 ± 0.051	0.055 ± 0.033	0.065 ± 0.014
Hippocampus	1.84 ± 0.38	17.5 ± 2.5	0.125 ± 0.022	0.153 ± 0.063	0.057 ± 0.036	0.079 ± 0.016
Precuneus	2.99 ± 0.69	28.4 ± 4.7	0.134 ± 0.025	0.146 ± 0.060	0.057 ± 0.035	0.076 ± 0.015
Putamen	3.34 ± 0.70	31.7 ± 4.2	0.136 ± 0.025	0.177 ± 0.075	0.054 ± 0.034	0.097 ± 0.019
Thalamus	2.77 ± 0.63	26.2 ± 4.0	0.114 ± 0.021	0.152 ± 0.055	0.053 ± 0.031	0.071 ± 0.011
Cerebellum	2.21 ± 0.50	20.9 ± 3.3	0.150 ± 0.030	0.202 ± 0.063	0.041 ± 0.021	0.083 ± 0.016

Results were obtained with the derived MDIFs and are presented as mean ± one standard deviation

*CMRGlu* Cerebral metabolic rate of glucose; *GM* Grey matter; *MDIF* Model-derived input function; *VOI* Volume-of-interest; *WM* White matter

## Discussion

This study investigated a SIME approach to generate an input function that replaced the commonly used series of exponentials by a model-derived equation based on the irreversible two-tissue compartment model. The simulations demonstrated that the input function can be recovered accurately by the SIME routine, with errors in macro- and microparameters of less than 1%. The accuracy of the MDIF approach predicted by the simulations was confirmed in the animal study in which the MDIF-to-AIF AUC ratio was close to unity and the input functions demonstrated good agreement (Fig. 3). The discrepancy between MDIF and AIF peaks could be partially explained by the assumption of a constant blood-to-plasma ratio of one, which might not be accurate in this animal model. For instance, Somogyi observed that the entirety of true sugars in the blood of pigs was present in plasma [41], although we could not find any studies that measured the blood-to-plasma ratio of [^18^F]FDG in this species. Future studies are needed to measure the [^18^F]FDG blood-to-plasma for this animal model.

Good agreement between MDIFs and IDIFs was observed when the two SIME approaches were applied to dynamic [^18^F]FDG PET data from healthy human participants (Fig. 4). No significant differences between ${K}_{i}$ or CMRGlu estimates were found, most likely since both methods used the same anchors. Since the MDIF is derived directly from the tissue TAC, and not restricted to gamma distribution functions as the original SIME, it is applicable to any injection protocol. The flexibility of the method is evident in Fig. 5. The injection protocol in this study involved a flush of saline that resulted in an inadvertent second peak during the early phase of the tissue TACs. This unexpected shape was only properly characterized by the MDIF method as evident by the improved fit of the tissue TACs to the 2TCM solution (Fig. 5C–F). A further advantage of the MDIF approach is the number of fitting parameters required to characterize the input function was reduced from seven (i.e., Eq. (6)) to four. The higher number of parameters needed to characterize the IDIF leads to multiple local minima during the minimization procedure, making the IDIF SIME procedure more sensitive to the chosen starting values. Consequently, higher variance of the estimated macroparameter (${K}_{i}$) was observed when deriving the IDIF with the SIME routine (CV of ~ 5%) compared to MDIF SIME (~ 2%).

As with all SIME methods, the MDIF approach requires TACs with varying kinetics and anchors to stabilize the minimization routine. The former was achieved using the *k*-means clustering algorithm, as previously suggested [42]. For the latter, either one or two blood samples were sufficient to properly capture the shape of the MDIF; however, the presence of noise in experimental PET data could introduce errors in the procedure. Care should be taken when selecting the appropriate number of anchors since previous studies observed that the use of a single scaling point was not sufficient to calibrate IDIFs [9, 18]. A potential limitation with anchors is that measurement errors will propagate through the SIME procedure. Increasing the number of venous samples could reduce these errors. In addition, consideration should be given when selecting the time-window to collect venous blood, as arterio-venous equilibrium occurs at different times for different tracers [43]. Although a completely non-invasive approach would be ideal, as proposed previously [23, 44], arterial or venous samples should always be considered to improve quantification accuracy.

The MDIF method has several advantages over other methods proposed to extract an IDIF. Compared to vessel segmentation approaches, it is free of PVEs and can produce input functions with greater SNR given the MDIF is derived from the WB TAC, while segmentation approaches often suffer from lower SNR due to the limited vessel volume used to extract the IDIF. Furthermore, IDIFs extracted from carotid arteries tend to overestimate their tails and scaling them with blood samples is recommended for quantification [15]. Additionally, the MDIF method can be used for any PET scanner given that the segmentation portion (i.e., *k*-means clustering; Fig. 4F) can be implemented by using a WB mask obtained from PET-only data. Thus, there is no need for specific anatomical data, such as time-of-flight MR images—often required in standard IDIF extraction methods. A further advantage of the MDIF approach is that it accounts for subject-specific variations in metabolism, which is a known limitation of population-based input functions. These advantages place the MDIF SIME approach at a unique position to be implemented in any PET centre. However, it may be necessary to incorporate a distributed parameter model to better character the vascular phase of the tracer considering recent advances in PET imaging are enabling the temporal resolution to be reduced to 1 or 2 s [45].

Although no arterial sampling was available to validate the MDIF SIME approach in humans, the CMRGlu values and rate constants obtained in the human study were in agreement with literature estimates [9, 35–37, 46–48] (Additional file 1: Table S1). Future studies involving human participants aimed at validating the MDIF SIME method would also provide the opportunity to optimize the technique by investigating the use of other parameter estimation methods—such as the simulated annealing method [49] implemented by Ogden et al. to minimize the SIME cost function [38]. As it is, the derived MDIF better characterized the first pass of [^18^F]FDG when compared to the IDIF modelled as a series of exponentials (Fig. 5).

The most obvious application of the MDIF would be to estimate CMRGlu from dynamic [^18^F]FDG PET data; however, there is growing interest in understanding how glucose metabolism is potentially affected by disease-related alterations in glucose delivery and phosphorylation [50, 51]. Altered microparameters have been observed in gliomas [52], suggesting they could improve differential diagnosis of malignant brain tumors [53]. Likewise, evidence of cerebrovascular contributions to Alzheimer’s disease has led to the hypothesis that hypometabolism characteristic of neurodegeneration is related, in part, to impaired glucose transport across the blood–brain barrier (i.e., ${K}_{1}$) [51]. These examples highlight the value of optimizing key steps in quantifying glucose metabolism to better understand major neurological diseases. However, accurately estimating the model rate constants at a voxelwise level is challenging [54]. Quantification of microparameters by kinetic modelling suffers from substantial statistical uncertainties due to the high noise level affecting voxel TACs. Efforts to develop image denoising approaches to improve quantification accuracy include post-reconstruction methods, such as the 4-dimensional iterative highly constrained backprojection algorithm [55], and during image reconstruction, such as physics-informed artificial intelligence reconstruction algorithms trained to increase the SNR [56]. Additionally, the accuracy of microparameters is linked to the choice of parameter estimation method [57, 58]. Lastly, the measured AIF, required for proper quantification, is an inherently noisy procedure that is often fit with a mathematical model to generate a continuous noiseless version for kinetic analysis [22]. The ability to generate high-quality input functions with the model-driven SIME approach could improve the reliability of microparameter estimates (Fig. 5), especially when combined with the variational Bayesian [33] parameter estimation method. In the parametric images obtained in this study (Fig. 7), ${K}_{1}$ maps presented the expected pattern of higher delivery of [^18^F]FDG to GM tissue.

Although a more sophisticated implementation is required, the MDIF SIME approach described here can be extended to other radiotracers that follow a reversible 2TCM (see Appendix), including when there are concerns regarding the irreversibility of [^18^F]FDG. Sari et al. implemented the IDIF SIME approach to evaluate dynamic data acquired with a serotonin receptor tracer and observed similar results in comparison to those obtained with the measured AIF [25]. Bartlett et al. and Ogden et al. used venous samples as scalers in the IDIF SIME method for a variety of radiotracers, including [^18^F]FDG, and reported good agreement to results obtained when arterial blood scalers were used, albeit with slightly biased estimates [38, 43]. Like with other SIME methods, only the plasma input function is recovered, while correcting for vascular contributions requires the whole-blood input function [38]. Further work is required to investigate if the MDIF method can be extended to simultaneously estimate the whole-blood input function while performing metabolite correction. Finally, similar to methods proposed for measuring cerebral blood flow [59, 60], an MDIF equation for radiotracers that follow the one-tissue compartment model could be derived following the steps outlined in the Appendix.

## Conclusion

In this study, we presented a SIME method based on the irreversible 2TCM to derive a high SNR input function. The extracted MDIF was compared to AIF curves in animal experiments and good MDIF-to-AIF agreement was observed. In the human study, MDIF macroparameters (i.e., ${K}_{i}$ and CMRGlu) estimates were in agreement with estimates obtained using the IDIF SIME method, which models the input function by a series of exponentials. Rate constants obtained with the MDIF were in good agreement with literature values. The MDIF approach has the advantages that it can model the shape of any input function and requires fewer fitting parameters. Additionally, combining the derived MDIF with the variational Bayesian approach allowed for the generation of parametric images of kinetic parameters.

### Supplementary Information

**Additional file 1.** Supplementary Figures and Tables.

## Data Availability

Scripts used in this study are available for download in an open repository (a link will be provided upon acceptance). The datasets generated and/or analysed during the current study are not publicly available due to data privacy but are available from the corresponding author on reasonable request.

## Appendix

The derivation of the MDIF equation (Eq. (4)) begins by combining the differential equations from the irreversible 2TCM (Eqs. (1) and (2)) after applying the Laplace Transform. Thus, the activity concentration in the first ($\overline{{C }_{1}}\left(s\right)$), second ($\overline{{C }_{2}}\left(s\right)$), and total ($\overline{{C }_{T}}\left(s\right)$) compartments are given by:

7 $$\overline{{C }_{1}}\left(s\right)=\frac{{K}_{1}}{s+{k}_{f}}\overline{{C }_{p}}\left(s\right)$$

8 $$\overline{{C }_{2}}\left(s\right)=\frac{{K}_{1}{k}_{3}}{s\left(s+{k}_{f}\right)}\overline{{C }_{p}}\left(s\right)$$

9 $$\overline{{C }_{T}}\left(s\right)=\overline{{C }_{1}}\left(s\right)+\overline{{C }_{2}}\left(s\right)=\left(1+\frac{{k}_{3}}{s}\right)\frac{{K}_{1}}{s+{k}_{f}}\overline{{C }_{p}}\left(s\right)$$

where $s$ is the Laplace variable. For this step, the following Laplace transform was used: ${L}\left\{{f}{\prime}\left(t\right)\right\}=s\overline{F }\left(s\right)-f\left(0\right)$, given ${C}_{1}\left(0\right)={C}_{2}\left(0\right)=0$.

As PET measurements ($\overline{{C }_{PET}}\left(s\right)$) include both tissue and blood activity contributions (i.e., ${C}_{PET}\left(t\right)=\left(1-{V}_{b}\right){C}_{T}\left(t\right)+{V}_{b}{C}_{b}\left(t\right)$), an additional term must be included to account for blood volume (${V}_{b}$) activity concentration originated from the whole-blood input function (${C}_{b}\left(t\right)=R{C}_{p}\left(t\right)$, where $R$ is the blood-to-plasma ratio):

10 $$\overline{{C }_{PET}}\left(s\right)=\left(1-{V}_{b}\right)\overline{{C }_{T}}\left(s\right)+{V}_{b}R\overline{{C }_{p}}\left(s\right)$$

Including Eqs. (9) in (10) yields:

11 $$\overline{{C }_{PET}}\left(s\right)=\left[\left(1-{V}_{b}\right)\left(1+\frac{{k}_{3}}{s}\right)\frac{{K}_{1}}{s+{k}_{f}}+R{V}_{b}\right]\overline{{C }_{p}}\left(s\right)$$

which can be rewritten as:

12 $$\overline{{C }_{PET}}\left(s\right)=\left[\frac{\left(s+{\alpha }_{1}\right)\left(s+{\alpha }_{2}\right)}{s\left(s+{k}_{f}\right)}R{V}_{b}\right]\overline{{C }_{p}}\left(s\right)$$

where ${\alpha }_{\mathrm{1,2}}=a\mp \sqrt{{a}^{2}-b}$, with $a=\frac{1}{2}\left(\frac{1-{V}_{b}}{R{V}_{b}}{K}_{1}+{k}_{f}\right)$, and $b=\frac{1-{V}_{b}}{R{V}_{b}}{K}_{1}{k}_{3}$.

Next, the MDIF can be obtained by isolating $\overline{{C }_{p}}\left(s\right)$ in Eq. (12), which requires partial fraction decomposition, and the resulting equation is given by:

13 $$\overline{{C }_{p}}\left(s\right)=\frac{1}{R{V}_{b}}\left[1+\frac{1-{V}_{b}}{R{V}_{b}}{K}_{1}\frac{1}{{\alpha }_{2}-{\alpha }_{1}}\left(\frac{{k}_{3}-{\alpha }_{2}}{s+{\alpha }_{2}}-\frac{{k}_{3}-{\alpha }_{1}}{s+{\alpha }_{1}}\right)\right]\overline{{C }_{PET}}\left(s\right)$$

The solution for ${C}_{p}^{MDIF}\left(t\right)$ given by Eq. (4) is obtained by applying the inverse Laplace Transform to Eq. (13), considering: ${{L}}^{-1}\left\{\frac{1}{s+a}\right\}={e}^{-at}$ and ${{L}}^{-1}\left\{\overline{F }\left(s\right)\bullet \overline{G }\left(s\right)\right\}=f\left(t\right)*g\left(t\right)$, where $*$ is the convolution operator.

The MDIF, ${C}_{p}^{MDIF}\left(t\right)$, for reversible radiotracers is obtained by solving for $\overline{{C }_{p}}\left(s\right)$ following the steps outlined above:

14 $${C}_{p}^{MDIF}\left(t\right)=\frac{1}{R{V}_{b}}\left[{C}_{wb}\left(t\right)+\frac{1-{V}_{b}}{R{V}_{b}}{K}_{1}\left(\frac{{k}_{3}+{k}_{4}-{\alpha }_{2}}{{\alpha }_{2}-{\alpha }_{1}}{e}^{-{\alpha }_{2}t}-\frac{{k}_{3}+{k}_{4}-{\alpha }_{1}}{{\alpha }_{2}-{\alpha }_{1}}{e}^{-{\alpha }_{1}t}\right)*{C}_{wb}\left(t\right)\right]$$

where ${\alpha }_{\mathrm{1,2}}=a\mp \sqrt{{a}^{2}-4b}$ $a=\frac{1}{2}\left(\frac{1-{V}_{b}}{R{V}_{b}}{K}_{1}+{k}_{f}+{k}_{4}\right)$, and $b=\frac{1-{V}_{b}}{R{V}_{b}}{K}_{1}\left({k}_{3}+{k}_{4}\right)+{k}_{2}{k}_{4}$. Note, here we considered radiotracers of constant blood-to-plasma ratio and no metabolite production. The inclusion of metabolite correction would require a more sophisticated solution, which is outside the scope of this work.

## Abbreviations

[^18^F]FDG: 2-Deoxy-2-[^18^F]fluoro-D-glucose
2TCM: Two-tissue compartment model
3D: 3-Dimensional
3D-OP-OSEM: 3D ordinary Poisson ordered subset expectation maximization
AIF: Arterial input function
a.u.: Arbitrary units
AUC: Area under the curve
CI: Confidence interval
CMRGlu: Cerebral metabolic rate of glucose
CT: Computed tomography
CV: Coefficient of variation
FoV: Field-of-view
GM: Grey matter
HYPR3D: 3D highly constrained backprojection
IDIF: Image-derived input function
MDIF: Model-derived input function
MNI: Montreal Neurological Institute
MPRAGE: Magnetization-prepared rapid gradient-echo
MRI: Magnetic resonance imaging
NLLS: Non-linear least squares
PET: Positron emission tomography
PVE: Partial volume effects
RSS: Residual sum of squares
SIME: Simultaneous estimation
SUV: Standardized uptake value
TAC: Time-activity curve
TE: Echo time
TR: Repetition time
WB: Whole brain
WM: White matter
WNLLS: Weighted non-linear least squares
WRSS: Weighted residual sum of squares
VOI: Volume-of-interest
