# Supplementary material for: Simultaneous estimation of a model-derived input function for quantifying cerebral glucose metabolism with [18F]FDG PET
Source: EJNMMI Phys. 2024 Jan 29;11:11. doi: 10.1186/s40658-024-00614-6 (PMC10825104; doi:10.1186/s40658-024-00614-6)
Supplement: Supplementary file 1 — Additional file 1. Supplementary Figures and Tables. [file 40658_2024_614_MOESM1_ESM.docx]

# Supplemental Material


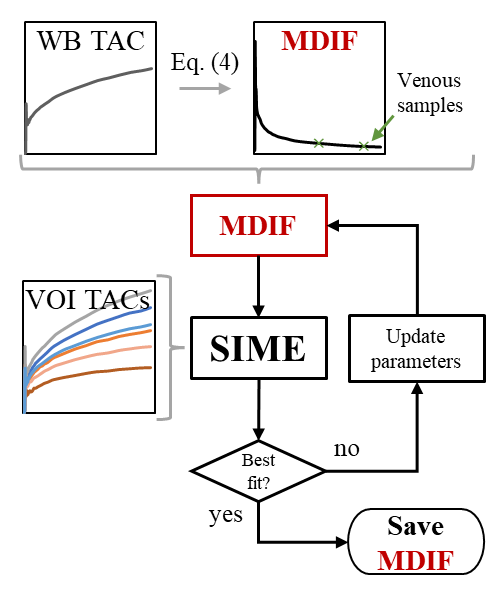


Supplemental Figure 1. MDIF SIME implementation diagram. The iterative process within the MATLAB optimization routine *lsqnonlin* starts by extracting the MDIF (Eq. (4)) from the WB TAC. Following, the MDIF is used to fit each VOI TAC to Eq. (3). Then, the parameters are updated, and the process is repeated until the cost function is minimized.


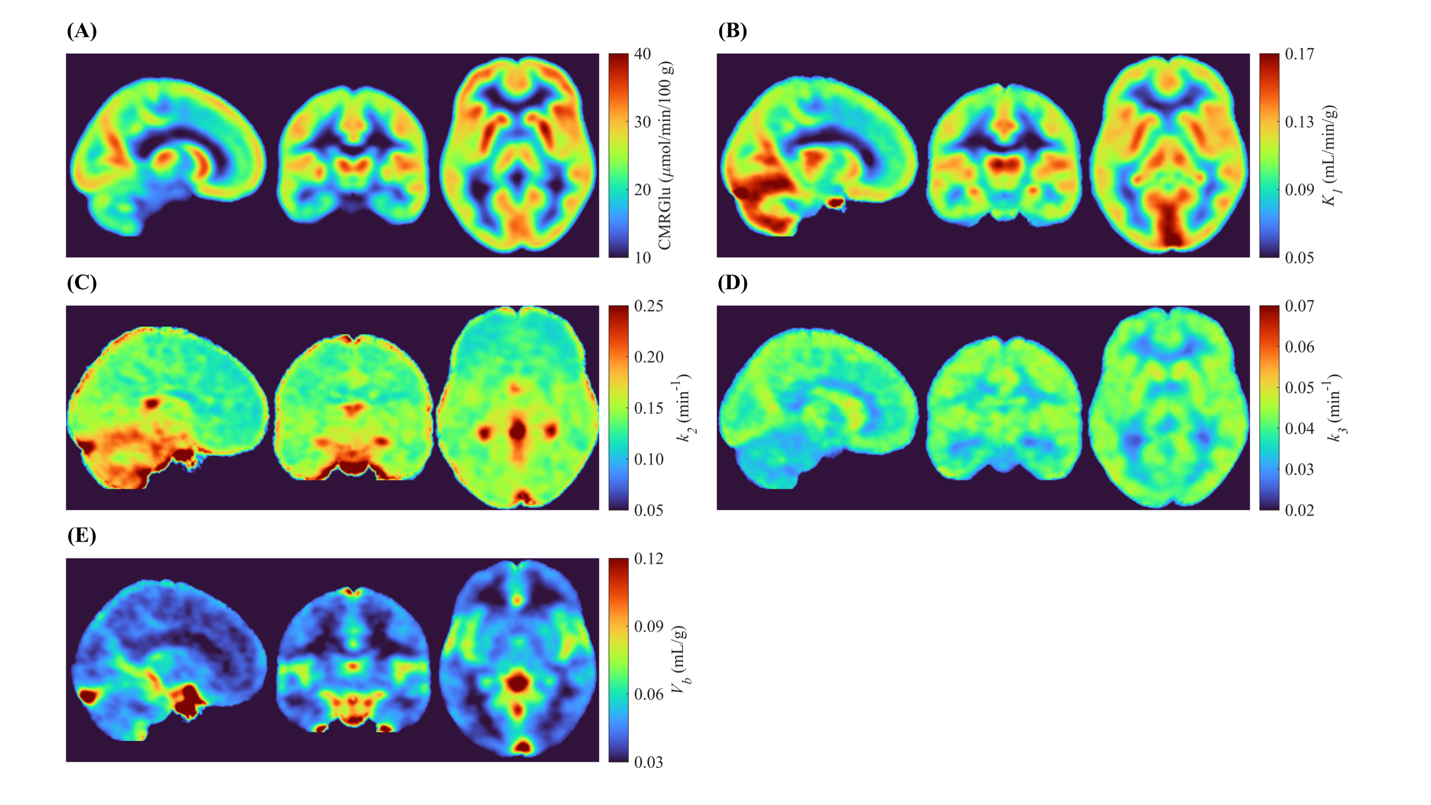


Supplemental Figure 2. Groupwise (*n* = 18) (A) CMRGlu (in µmol/100 g/min) images, alongside (B) $K_{1}$ (in mL/g/min), (C) $k_{2}$ (in min^−1^), (D) $k_{3}$ (in min^−1^), and (E) $V_{b}$ (in mL/100 g) images, all from the variational Bayesian fitting routine. All images were generated using the IDIFs and normalized to the MNI space with SPM12.


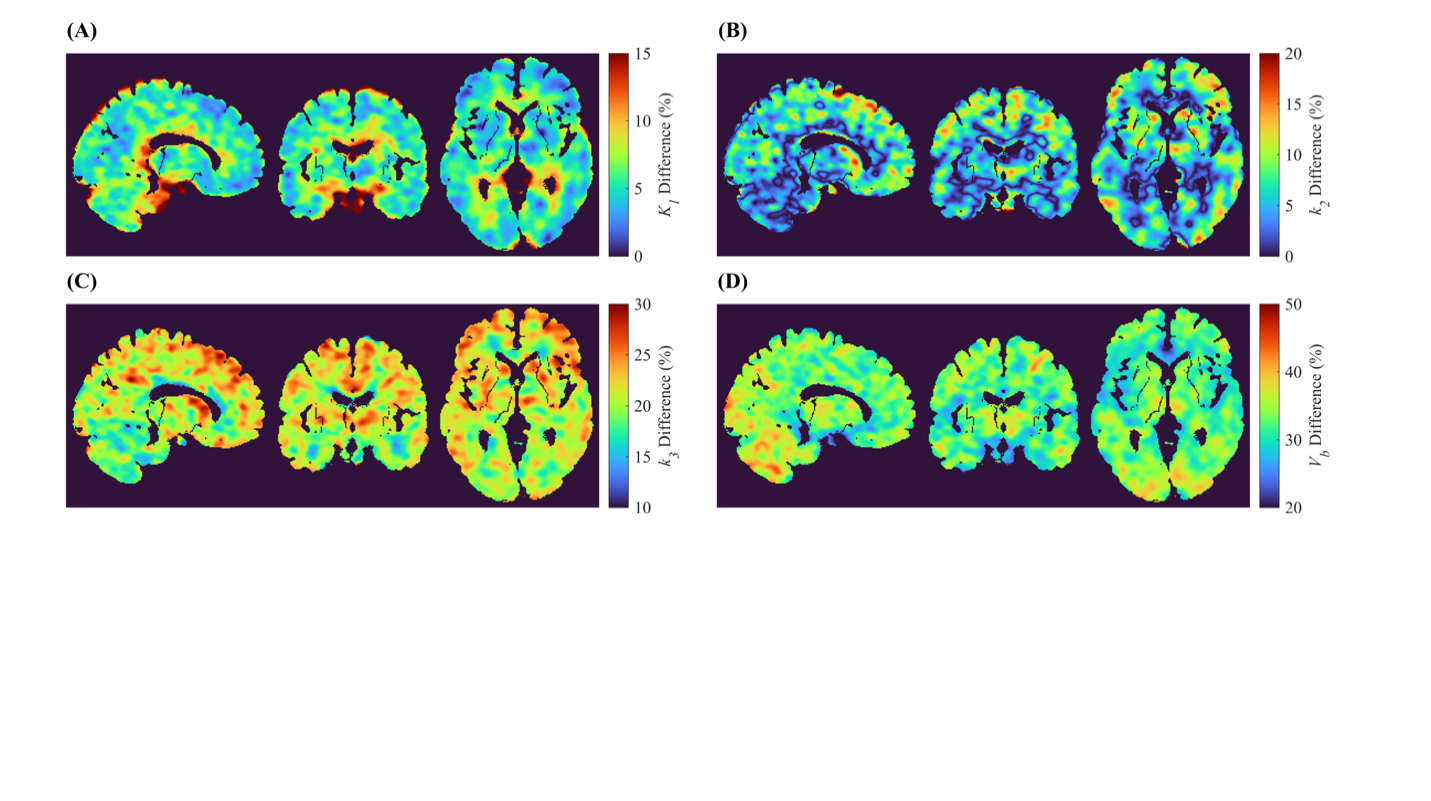


Supplemental Figure 3. Groupwise (*n* = 18) difference (%) (A) $K_{1}$, (B) $k_{2}$, (C) $k_{3}$, and (D) $V_{b}$ images obtained with the MDIFs and IDIFs as inputs into the variational Bayesian fitting routine. All images were normalized to the MNI space with SPM12.

Supplemental Table 1. GM $K_{i}$, CMRGlu, and rate constants reported in the literature for healthy individuals. GM CBV is also included when available. Between-subject variability, estimated by the coefficient of variation (i.e., standard deviation divided by the average measurement) is shown in brackets when available. Arterial sampling was used to measure the input function for all studies, unless otherwise specified. Reported CMRGlu were converted to a lumped constant of 0.52 when possible. Values are displayed in terms of mean ± one standard deviation.

|  | $\boldsymbol{K}_{\boldsymbol{i}}$  (mL/100 g/min) | CMRGlu  (µmol/100 g/min) | $\boldsymbol{K}_{\boldsymbol{1}}$  (mL/g/min) | $\boldsymbol{k}_{\boldsymbol{2}}$  (min^−1^) | $\boldsymbol{k}_{\boldsymbol{3}}$  (min^−1^) | $\boldsymbol{V}_{\boldsymbol{b}}$  (mL/g) |
| --- | --- | --- | --- | --- | --- | --- |
| Present study (*n* = 18) ^a^ | 2.46 ± 0.53  (22%) | 24.5 ± 3.6  (15%) | 0.120 ± 0.022  (19%) | 0.160 ± 0.057  (36%) | 0.051 ± 0.029  (57%) | 0.072 ± 0.013  (18%) |
| Sari *et al*. (2017) [9] (*n* = 19) | 4.9 ± 0.4  (8%) | 46.0 ± 4.0  (9%) | 0.123 ± 0.014  (11%) | 0.121 ± 0.045  (37%) | 0.079 ± 0.023  (29%) | 0.059 ± 0.019  (32%) |
| Huisman *et al*. (2012) [37] (*n* = 9) | 3.1 ± 0.4  (13%) | 29 ± 3  (10%) | 0.062 ± 0.008  (13%) | 0.071 ± 0.040  (56%) | 0.067 ± 0.030  (45%) | 0.05 ± 0.01 ^b^  (20%) |
| Zanotti-Fregonara *et al*. (2009) [36] (*n* = 4) ^c^ | 2.7 ^d^ | NA | 0.096 ± 0.020  (21%) | 0.109 ± 0.012  (11%) | 0.042 ± 0.001  (2%) | NA |
| Mosconi *et al*. (2007) [48] (*n* = 6) ^e^ | 3.8 ± 0.7  (18%) | 42.8 ± 2.3  (18%) | 0.14 ± 0.02  (14%) | 0.37 ± 0.06  (16%) | 0.14 ± 0.03  (21%) | NA |
| Sasaki *et al*. (1986) [47] (*n* = 7) ^f^ | 3.1 ^d^ | 35.6 ± 4.0  (11%) | 0.074 ± 0.009  (13%) | 0.069 ± 0.022  (32%) | 0.051 ± 0.014  (27%) | NA ^g^ |
| Reivich *et al*. (1985) [35] (*n* = 9) ^h^ | 3.5 ^d^ | 31.4 ± 5.0 ^i^  (16%) | 0.105 ± 0.018  (17%) | 0.148 ± 0.024  (16%) | 0.074 ± 0.015  (20%) | NA |
| Huang *et al*. (1980) [46] (*n* = 13) ^j^ | 3.3 ± 0.6  (18%) | 32.6 ± 5.3  (17%) | 0.102 ± 0.028  (27%) | 0.130 ± 0.066  (51%) | 0.062 ± 0.019  (31%) | NA |

2TCM: two-tissue compartment model; CBV: cerebral blood volume; CMRGlu: cerebral metabolic rate of glucose; GM: grey matter; N/A: not applicable; SD: standard deviation; SE: standard error.

^a^ MDIF was used instead of arterial sampling.

^b^ VOI from which CBV was reported was not specified.

^c^ VOI from which rate constants were reported was not specified.

^d^ Calculated based on average rate constants.

^e^ Occipital love was used as representative of GM.

^f^ Left frontal lobe was used here as representative of GM.

^g^ CBV was corrected with [^15^O]CO-PET.

^h^ Standard error (SE) was converted to standard deviation (SD) as $SD=SE\sqrt{n}$.

^i^ CMRGlu was reported for whole brain (*n* = 6) and had units of mL/100 g/min, which were converted to µmol/100 g/min.

^j^ Reversible 2TCM model was applied; average $k_{4}$ estimates for GM were 0.0068 ± 0.0014

Supplemental Table 2. Microparameters from the two SIME approaches (*n* = 18). Results are presented as mean ± one standard deviation.

| Cluster | $\boldsymbol{K}_{\boldsymbol{1}}$ (mL/g/min) | $\boldsymbol{k}_{\boldsymbol{2}}$ (min^−1^) | $\boldsymbol{k}_{\boldsymbol{3}}$ (min^−1^) | $\boldsymbol{V}_{\boldsymbol{b}}$(mL/g) | *CV* (%) |
| --- | --- | --- | --- | --- | --- |
| MDIF | | | | | |
| WB^a^ | 0.103 ± 0.019 | 0.153 ± 0.055 | 0.050 ± 0.029 | 0.067 ± 0.018 | 0.4 ± 0.1 |
| GM #1 | 0.116 ± 0.026 | 0.161 ± 0.062 | 0.048 ± 0.026 | 0.072 ± 0.012 | 1.7 ± 1.3 |
| GM #2 | 0.122 ± 0.030 | 0.153 ± 0.058 | 0.052 ± 0.031 | 0.071 ± 0.017 | 1.7 ± 0.5 |
| GM #3 | 0.129 ± 0.023 | 0.144 ± 0.053 | 0.056 ± 0.033 | 0.074 ± 0.016 | 1.8 ± 0.5 |
| WM #1 | 0.063 ± 0.015 | 0.145 ± 0.054 | 0.041 ± 0.025 | 0.048 ± 0.008 | 3.6 ± 0.9 |
| WM #2 | 0.081 ± 0.017 | 0.150 ± 0.055 | 0.045 ± 0.026 | 0.054 ± 0.010 | 2.3 ± 0.6 |
| WM #3 | 0.096 ± 0.019 | 0.151 ± 0.056 | 0.049 ± 0.028 | 0.057 ± 0.011 | 1.6 ± 0.4 |
| IDIF | | | | | |
| WB^b^ | 0.109 ± 0.024 | 0.145 ± 0.048 | 0.040 ± 0.012 | 0.043 ± 0.010^c^ | 4.9 ± 2.0 |
| GM #1 | 0.127 ± 0.034 | 0.164 ± 0.066 | 0.041 ± 0.012 | 0.053 ± 0.016^c^ | 5.0 ± 2.1 |
| GM #2 | 0.130 ± 0.030 | 0.151 ± 0.051 | 0.044 ± 0.015 | 0.051 ± 0.013^c^ | 5.2 ± 1.9 |
| GM #3 | 0.138 ± 0.032 | 0.142 ± 0.055 | 0.046 ± 0.014 | 0.054 ± 0.014^c^ | 5.2 ± 2.0 |
| WM #1 | 0.073 ± 0.020 | 0.163 ± 0.062 | 0.037 ± 0.011 | 0.035 ± 0.008^c^ | 5.9 ± 1.8 |
| WM #2 | 0.090 ± 0.022 | 0.159 ± 0.060 | 0.039 ± 0.011 | 0.039 ± 0.009^c^ | 5.1 ± 1.7 |
| WM #3 | 0.105 ± 0.024 | 0.153 ± 0.058 | 0.042 ± 0.012 | 0.041 ± 0.009^c^ | 4.9 ± 1.8 |

IDIF: image-derived input function; GM: grey matter; MDIF: model-derived input function; SIME: simultaneous estimation; VOI: volume-of-interest; WB: whole brain; WM: white matter.

^a^ Estimates used to derive the MDIF with Eq. (4).

^b^ Shown for comparison as the WB TAC was not included in the IDIF SIME.

^c^ Significantly different than respective MDIF estimates (*p* < 0.05).
